# Characterizing the Lymphopoietic Kinetics and Features of Hematopoietic Progenitors Contained in the Adult Murine Liver In Vivo

**DOI:** 10.1371/journal.pone.0076762

**Published:** 2013-10-09

**Authors:** Xiaojun Jiang, Yongyan Chen, Haiming Wei, Rui Sun, Zhigang Tian

**Affiliations:** 1 Department of Immunology, School of Life Sciences, University of Science and Technology of China, Hefei, Anhui, China; 2 Hefei National Laboratory for Physical Sciences at Microscale, Hefei, Anhui, China; Rutgers – New Jersey Medical School, United States of America

## Abstract

The appearance of donor-derived lymphocytes in liver transplant patients suggests that adult livers may contain cells capable of lymphopoiesis. However, only a few published studies have addressed the lymphopoietic capacity of adult liver cells, and its kinetics and features remain unclear. Herein, we investigated the lymphopoietic capacity of adult liver mononuclear cells (MNCs) and purified liver hematopoietic progenitor cells (HPCs) in vivo. Similar to bone-marrow transplantation (BMT), transplantation of liver MNCs alone was able to rescue survival of lethally irradiated mice. In terms of kinetics, liver MNC-derived myeloid lineage cells reconstituted more slowly than those from BMT. Liver MNC-derived lymphocyte lineage cells in the blood, spleen and BM also reconstituted more slowly than BMT, but lymphocytes in the liver recovered at a similar rate. Interestingly, liver MNCs predominantly gave rise to CD3^+^CD19^−^ T cells in both irradiated WT and non-irradiated lymphocyte-deficient *Rag-1^−/−^Il2rg^−/−^* recipients. To define the lymphopoietic potential of various cell populations within liver MNCs, we transplanted purified lineage-negative (Lin^−^) liver HPCs into recipient mice. Unlike total liver MNCs, liver HPCs reconstituted T and B cells in similar frequencies to BMT. We further determined that the predominance of T cells observed after transplanting total liver MNCs likely originated from mature T cells, as purified donor liver T cells proliferated in the recipients and gave rise to CD8^+^ T cells. Thus, the capacity of donor adult liver cells to reconstitute lymphocytes in recipients derives from both HPCs and mature T cells contained in the liver MNC population.

## Introduction

Hematopoiesis is a basic physiological process required throughout the life of an individual. Since most mature blood cells are short-lived, replenishing hematopoietic cell-derived lineages from stem cells is required [1]. In general, the hematopoietic system originates from hematopoietic stem cells (HSCs) and hematopoietic progenitor cells (HPCs) that differentiate into two major lineages of mature hematopoietic cells: myeloid and lymphoid cells [2]. In mammals, hematopoiesis occurs in discrete niches that change frequently during ontogeny [3], [4]. Sequentially, blood cells are first produced in the yolk sac [5], [6], followed by the developing aorta-gonad-mesonephros region [7], [8], then the fetal liver [9], and finally the bone marrow (BM).

Although HSCs are generally considered to migrate from fetal liver to the BM during development, there is evidence to suggest that cells residing in the adult liver also have some hematopoietic capacity. This ability of the adult liver remains of great interest, especially in the transplantation field in which liver-derived hematopoiesis was first observed [10]. In many liver transplant recipients, donor blood chimerism is maintained for many years after successful solid organ transplantation, raising the possibility that hematopoietic cells exist in the transplanted livers [11]–[13]. In vitro experiments confirmed that adult liver cells harvested from both mice and humans could efficiently form hematopoietic colonies [14], [15]. Moreover, c-kit^+^Sca-1^+^Lin^lo/−^ cells as well as CD45^+^ liver side population tip cells were identified in adult livers; when transplanted into recipient mice, these cell populations demonstrated the ability to rescue the survival of lethally irradiated mice and to mediate reconstitution of multiple blood cell lineages [16]–[18]. These observations and experimental results provide strong evidence of the existence of HSCs and HPCs in the adult liver.

Although these cells have been identified and were determined to function as hematopoietic cells, the precise details of liver hematopoiesis are still unclear. While donor- derived cells have been traced by CD45.1 markers in a previous study [16], the subsequent dynamic changes within each of the resulting mature cell lineages were not characterized. Moreover, the lymphopoietic features of cells derived from HPCs, such as the various lymphoid cell subsets and their phenotypes, have not yet been well described. Additionally, it has been shown that donor blood chimerism in liver transplantation is derived not only from liver HPCs but also from mature cells [19], [20]; however, the relative contribution of those mature cells to generating liver-resident lymphocytes is also not well understood.

In this study, we described the kinetics and characteristics of lymphoid reconstitution by transplanting donor liver mononuclear cells (MNCs) into recipient mice in the same manner as BM transplantation (BMT). We subsequently studied the dynamic changes in, and reconstitution of, lymphoid lineage subsets after transplanting liver HPCs and compared them to cells derived from competing BM cells. Our results demonstrated that adult liver contains HPCs with lymphopoietic capacity, similar to those found in BM, as well as a dominant mature T cell population that could help to repopulate recipient livers. We also found that transplanting purified liver T cells predominantly gave rise to CD8^+^ T cells in the livers of recipient mice.

## Materials and Methods

### Mice

All experiments involving the use of mice were approved by the Animal Care and Use Committee at the University of Science and Technology of China. 8- to10-week-old C57BL/6 (B6) mice were purchased from the Shanghai Experimental Animal Center (Shanghai, China). CD45.1^+^ B6.SJL mice were purchased from the Jackson Laboratory (Bar Harbor, ME). CD45.1×CD45.2 and *Rag-1* ^−/−^ *Il2rg* ^−/−^ mice were generated and bred in-house.

### Ethics Statement

Mice were maintained in a specific pathogen-free facility according to the guidelines for experimental animals from the University of Science and Technology of China. The protocol was approved by Animal Care and Use Committee of University of Science and Technology of China (Permit Number: USTCACUC1201008). For survival study, mice were monitored every day. When mice appeared to be in distress as judged by independent animal care personnel with no knowledge of the protocol design, animals were euthanized by CO_2_. All surgeries were performed under sodium pentobarbital anesthesia, and all efforts were made to minimize suffering.

### Cell preparation

Livers were perfused, and liver suspensions were passed through a 200-gauge stainless steel mesh. Cells were resuspended in 40% Percoll (GE Healthcare, Uppsala, Sweden) and centrifuged at 1260×*g* for 15 min to remove debris. Liver MNCs were collected after lysing erythrocytes. Splenocytes were obtained by forcing the spleen through a 200-gauge stainless steel mesh and subsequently lysing erythrocytes. BM cells were isolated by flushing femurs and then lysing erythrocytes.

### Transplantation

Wild-type (WT) B6 mice were lethally irradiated by administering 1100 rads, and donor cells were intravenously transplanted 1 day later. Mice were maintained on a standard chow diet and water supplemented with sulfamethoxazole/trimethoprim (Sulfatrim) for 21 days. For *Rag-1^−/−^Il2rg^−/−^* mice, donor cells were intravenously transplanted without irradiation.

### Antibody staining and flow cytometry

The following antibodies were purchased from BD Biosciences (San Jose, CA): FITC-labeled anti-CD19, anti-CD25, anti-CD34, anti-CD62L; PE-labeled anti-CD3e, anti-CD45.1, anti-CD69, anti-CD117; PerCP-Cy5.5-labeled anti-CD3, anti-CD8a, anti-CD19, anti-CD44, anti Lineage Antibody Cocktail; APC-labeled anti-CD3e, anti-CD11c, anti-CD45.2, anti-CD117; APC-Cy7-labeled anti-CD3, anti-CD4, anti-CD45.1; and PE-Cy7-labeled anti-Sca-1, anti-NK1.1, anti-CD45.1. Cells were incubated with rat immunoglobulin for 30 min to block Fc receptors before staining with antibodies. We performed flow cytometry on an LSRII (BD Biosciences) and analyzed data using FlowJo software (TreeStar, Ashland, OR).

### Analysis of cell subsets in peripheral blood

Blood was collected from mice into EDTA-containing anticoagulation tubes and then analyzed by XT-1800i Automated Hematology Analyzer (Sysmex), which determined the number and frequency of white blood cells (WBCs), lymphocytes, neutrophils, eosinophils, monocytes, red blood cells (RBCs), and platelets (PLTs).

### Cell sorting

A FACSAria Cell Sorter (BD Biosciences) was used to purify Lin^−^ (CD3^−^CD19^−^NK1.1^−^CD11b^−^) precursors and CD3^+^ T cells. The purity of the sorted cell populations was >95% as assessed by flow cytometry.

### Statistical Analysis

We analyzed the data obtained from individual mice using the two-tailed unpaired Student's *t*-test or ANOVA. We performed log rank test on the survival data. Differences achieving values of *p*<0.05 were considered significant.

## Results

### Adult murine liver MNCs rescued lethally irradiated mice

**Figure 1:**
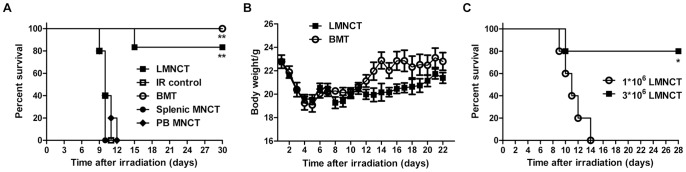
Lethally irradiated mice were rescued by liver MNC transplantation. (A) Survival curves of lethally irradiated B6 mice that received the indicated cells: 4×10^6^ liver MNCs (LMNCT), BMCs (BMT), splenic cells (splenic MNCT), or PBMCs (PB MNCT) from 0.3 mL total peripheral blood; lethally irradiated mice without transplantation served as the control group (n = 5–6 mice/group). (B) Weight changes in mice were monitored for over 3 weeks after liver MNC or BM transplantation. Data are represented as the mean ± SEM (n = 3–6 mice/group). (C) Survival of lethally irradiated B6 mice received the indicated number of liver MNCs (n = 5 per group). *p<0.05, **p<0.01. All experiments were repeated in no less than 2 independent experiments.

Donor-derived blood chimerism develops in liver transplant recipients, providing evidence that adult liver contains cells with hematopoietic potential. Liver grafts are mainly composed of hepatocytes and MNCs; since hepatocytes cannot generate blood cells, we hypothesize that progenitor cells likely came from the liver MNC population. To test this, we transplanted liver MNCs into lethally irradiated mice and evaluated their ability to rescue mouse survival compared to BMT. Similar to BM cells, liver MNCs rescued a high percentage of lethally irradiated mice almost 90% 1 month after transfer. In contrast, neither splenic MNC nor peripheral blood MNC (PBMCs) transplantation could rescue recipient mice, which all died within 12 days of irradiation (Fig. 1A). Thus, these results suggest that MNCs from liver, but not from spleen or blood, contain cells that can repopulate the hematopoietic system, rescuing mice from death by lethal irradiation. In accordance with the survival results, the body weights of mice receiving liver MNCs rapidly recovered and were maintained, also similar to BMT recipients (Fig. 1B). In addition, we observed a dose-dependent effect of the number of transplanted liver MNCs on mouse survival (Fig. 1C); from this result, we decided to use 3–4×10^6^ donor liver MNCs per mouse in subsequent experiments.

### Myeloid lineages were reconstituted by transplanting liver MNCs

**Figure 2:**
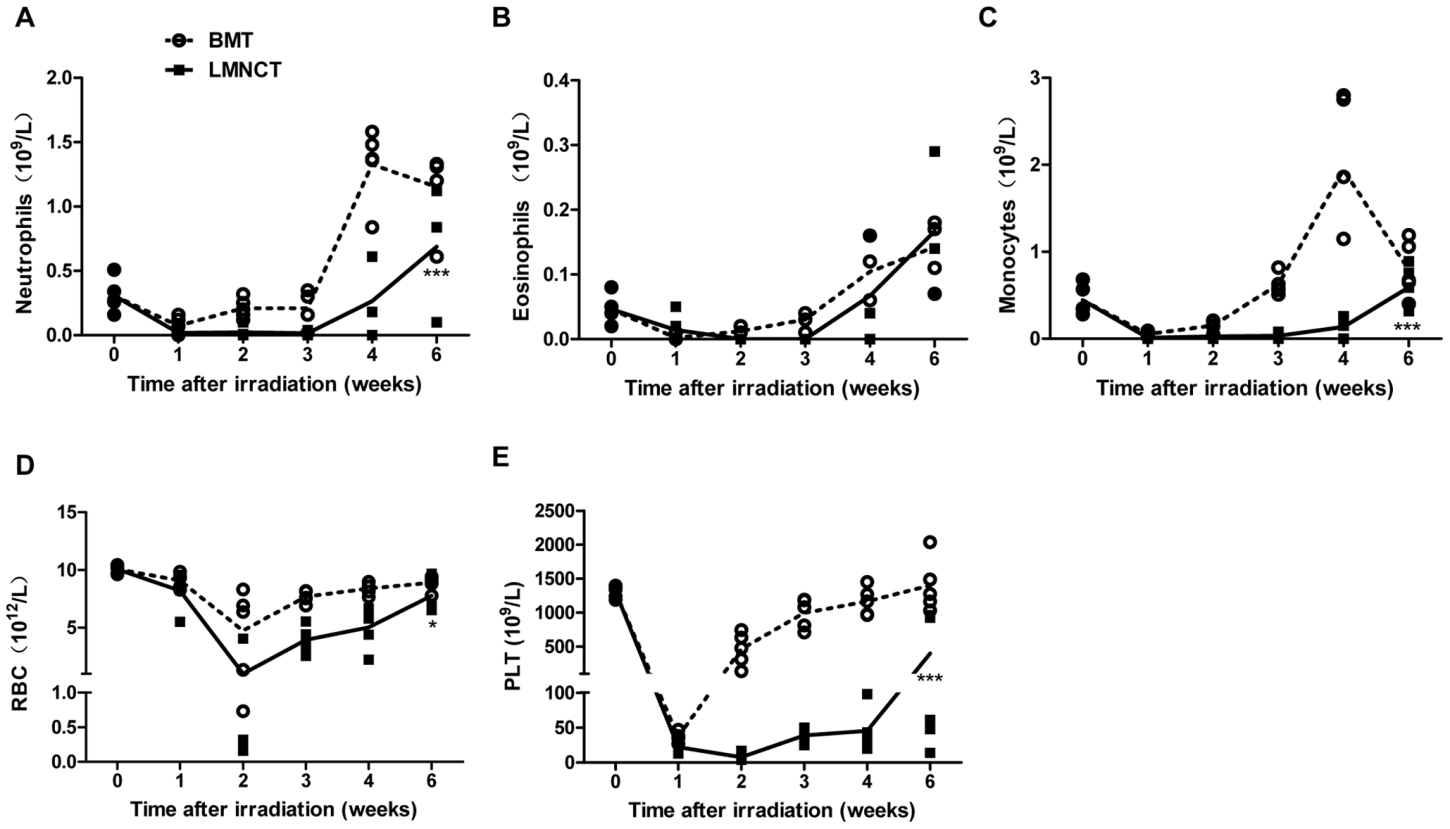
Myeloid lineage cells differentiated from donor liver MNCs were characterized in lethally irradiated recipients. Peripheral blood from B6 mice that received liver MNCs or BMCs after lethal irradiation was collected at the indicated time points, and dynamic changes in the number of (A) neutrophils, (B) eosinophils, (C) monocytes, (D) red blood cells (RBCs), and (E) platelets (PLTs) were tracked over 6 weeks. Each symbol represents an individual mouse, and the connected lines represent the mean cell numbers found for each experimental group. *p<0.05, ***p<0.001. Data were collected from 3 independent experiments.

In general, hematopoietic cells can reconstitute both the myeloid and lymphoid lineage compartments. We first examined whether liver MNC transplantation could reconstitute myeloid lineage cells, including neutrophils, eosinophils, monocytes, RBCs, and PLTs, compared to BMT. As shown in Fig. 2, myeloid lineage cells from liver MNCs were reconstituted 6 weeks post-transplant, which occurred more slowly than after BMT, suggesting that the hematopoietic capacity of liver MNCs to repopulate the myeloid cell lineage is weaker than that of BM cells.

### The liver MNC-reconstituted immune system was dominated by T cells

**Figure 3:**
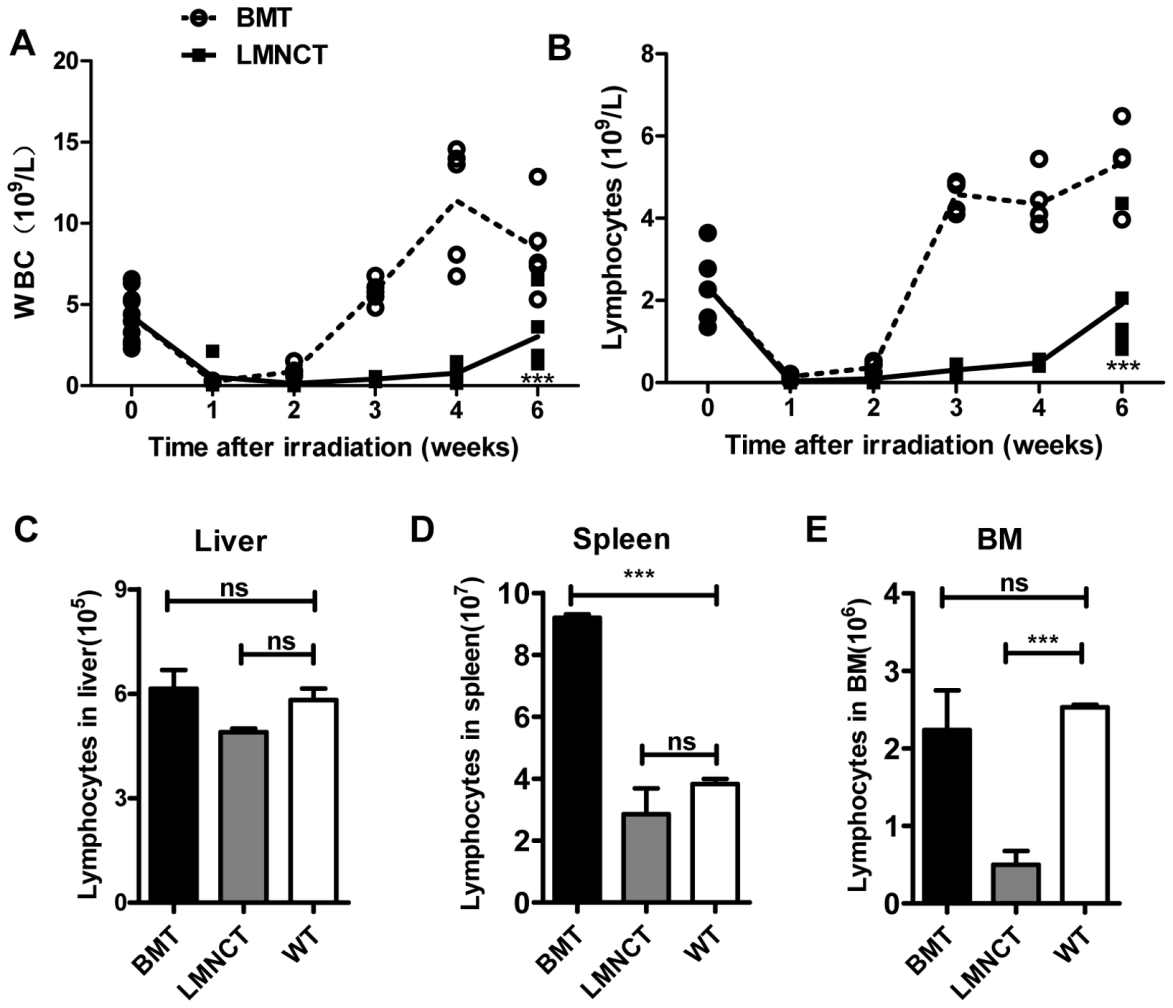
Lymphoid lineage cells reconstituted after liver MNCs transplantation were characterized. The absolute number of (A) WBCs or (B) lymphocytes in the peripheral blood of recipients after liver MNC or BM transplantation were tracked over 6 weeks (n = 5 mice/group). (C–E) Absolute number of lymphocytes in (C) liver, (D) spleen, and (E) BM was calculated 3 weeks after BMT or liver MNC transplantation (LMNCT). Wild-type (WT) mice without irradiation served as the control group. Data are represented as the mean ± SEM (n = 2–3 mice/group). ***p<0.001. All data are representative of no less than 2 independent experiments.

We next investigated the ability of liver MNCs to reconstitute lymphoid lineage cells in the peripheral blood of recipients as compared to BM. In contrast to the robust increase in peripheral white blood cells (WBCs) and lymphocytes after BMT, the recovery of WBCs or lymphocytes after transplanting liver MNCs was relatively slow (Fig. 3A–B), similar to the reconstitution of myeloid cells. To determine the lymphocyte lineage reconstitution in various tissues of the recipients, we isolated MNCs from liver, spleen, and BM 1 month after transplanting liver MNCs and compared the frequency of lymphoid subsets with that of BMT recipients as well as with normal mice that did not receive irradiation. Interestingly, the recovery of recovered lymphocytes varied among the tissues. Notably, the absolute number of hepatic lymphocytes was not different among the 3 experimental groups (Fig. 3C). In the spleen, liver MNCs gave rise to lymphocyte numbers that were similar to normal mice, whereas these numbers were markedly increased by several fold after BMT (Fig. 3D). In the BM, however, liver MNCs were not able to recover the lymphocyte numbers observed in normal mice or after BMT (Fig. 3E). Thus, these data indicate that liver MNCs reconstitute lymphocyte lineage cells more slowly than BM cells, but are capable of eventually reconstituting normal lymphocyte numbers in the liver and spleen, but not BM, of recipient mice.

**Figure 4:**
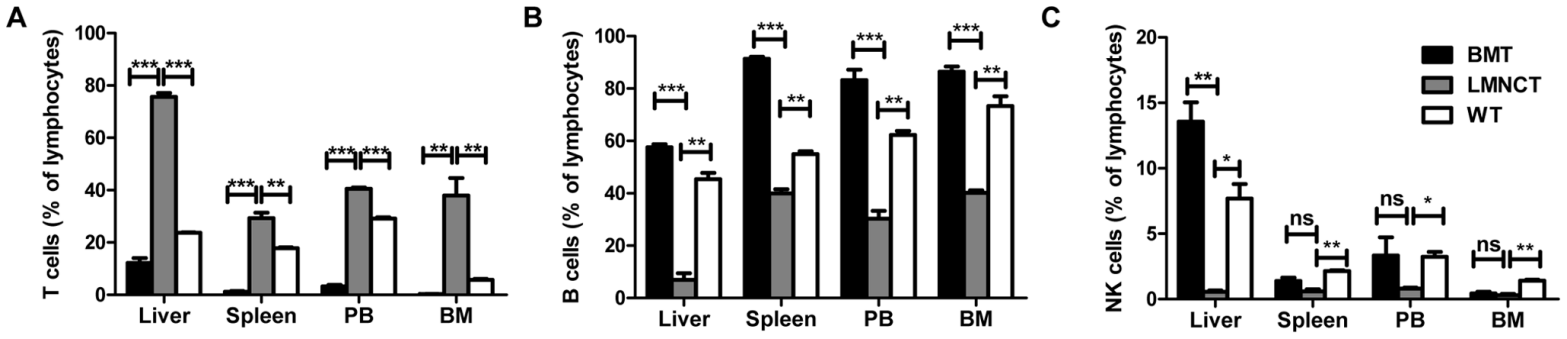
Lymphoid lineage subsets reconstituted after liver MNC transplantation were characterized. The percentage of (A) CD3^+^CD19^−^ T cells, (B) CD3^−^CD19^+^ B cells, or (C) CD3^−^CD19^−^NK1.1^+^ NK cells among total lymphocytes in spleen, liver, peripheral blood (PB), and BM were shown in recipient mice 3 weeks after BMT and liver MNC transplantation. Data are represented as the mean ± SEM (n = 2–3 mice/group). *p<0.05, **p<0.01, ***p<0.001. All data are representative of no less than two independent experiments.

To identify and determine the proportions of the various lymphoid cell types that arose from liver MNCs 1 month after transplantation, we analyzed the lymphocyte subpopulations in several tissues and compared them to BMT. Whereas most of the repopulated cells after BMT were CD3^−^CD19^+^ B cells (Fig. 4B), we found that liver MNCs repopulated much more CD3^+^CD19^−^ T cells in recipient mice (Fig. 4A) and that CD3^−^CD19^−^NK1.1^+^ NK cells were rare in all examined organs of liver MNC recipients (Fig. 4C). These results suggest that while BM cells preferentially repopulate B cells, liver MNCs preferentially repopulate T cells.

**Figure 5:**
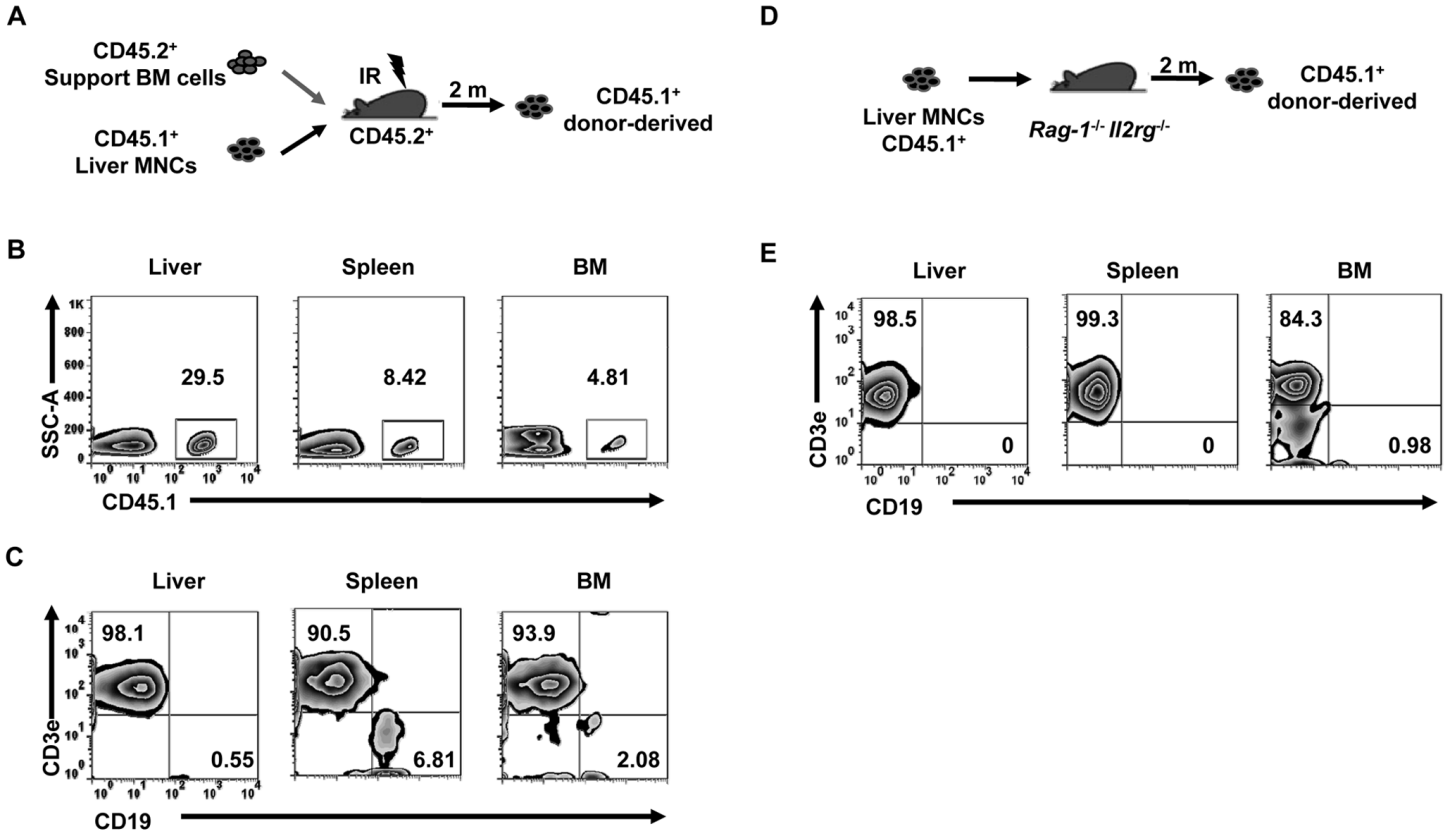
Survival of liver MNC-derived cells in lethally irradiated mice. (A) Hepatic MNCs (10^6^) from CD45.1^+^ mice were mixed with 10^6^ support BM cells from CD45.2^+^ mice and transferred into lethally irradiated CD45.2^+^ B6 mice. (B–C) Two months post-transfer, CD45.1^+^ cells in the recipient liver, spleen, and BM were gated, and the expression of CD3 and CD19 was analyzed (n = 3 mice/group). (D) Hepatic MNCs (10^6^) from CD45.1^+^ mice were transferred into CD45.1^−^ *Rag-1^−/−^Il2rg^−/−^* mice. (E) Two months post-transfer, CD45.1^+^ donor cells in the recipient liver, spleen, and BM were gated, and the expression of CD3e and CD19 were analyzed (n = 3 mice/group). All data are representative of 2 independent experiments.

Since lethal irradiation could not completely delete host-derived HSCs, it remained possible that the observed reconstituted cell populations were derived from host cells. Thus, to distinguish between donor-derived cells and recipient-derived cells, we used liver MNCs from CD45.1^+^ mice to track donor-derived leukocytes by flow cytometry (Fig. 5A). Since it was difficult to obtain sufficient numbers of donor cells because of the limited availability of CD45.1 mice for this experiment, we co-transplanted CD45.1^+^ liver MNCs along with CD45.2^+^ support BM cells to improve recipient survival. Interestingly, CD45.1^+^ liver-derived lymphocytes preferentially resided in recipient livers, as CD45.1^+^ cells constituted nearly 30% of total liver lymphocytes 2 months after transfer, while their frequency in spleen and BM was only around 5% (Fig. 5B). Consistent with our previous experiments, T cells were the dominant liver MNC-derived lymphocyte population in the various tissues of the recipients in these experiments (Fig. 5C).

Lethal irradiation may also lead to variations within the microenvironment, which could possibly affect the outcome of lymphopoiesis. Thus, we also examined liver MNC-derived lymphopoiesis in *Rag-1* ^−/−^ *Il2rg* ^−/−^ recipient mice (Fig. 5D), as they lack almost all lymphocytes and can accept donor cells without irradiation. Two months after liver MNC transplantation, we again observed that liver MNCs were able to reconstitute a T cell-dominated immune system in these non-irradiated recipients (Fig. 5E), similar to liver MNCs in irradiated WT mice. We therefore conclude from these 2 independent models of hematopoiesis that donor liver MNCs can repopulate the mature lymphocytes found in recipient mice.

### Liver MNCs contained HPCs that were functionally similar to BM cells

**Figure 6:**
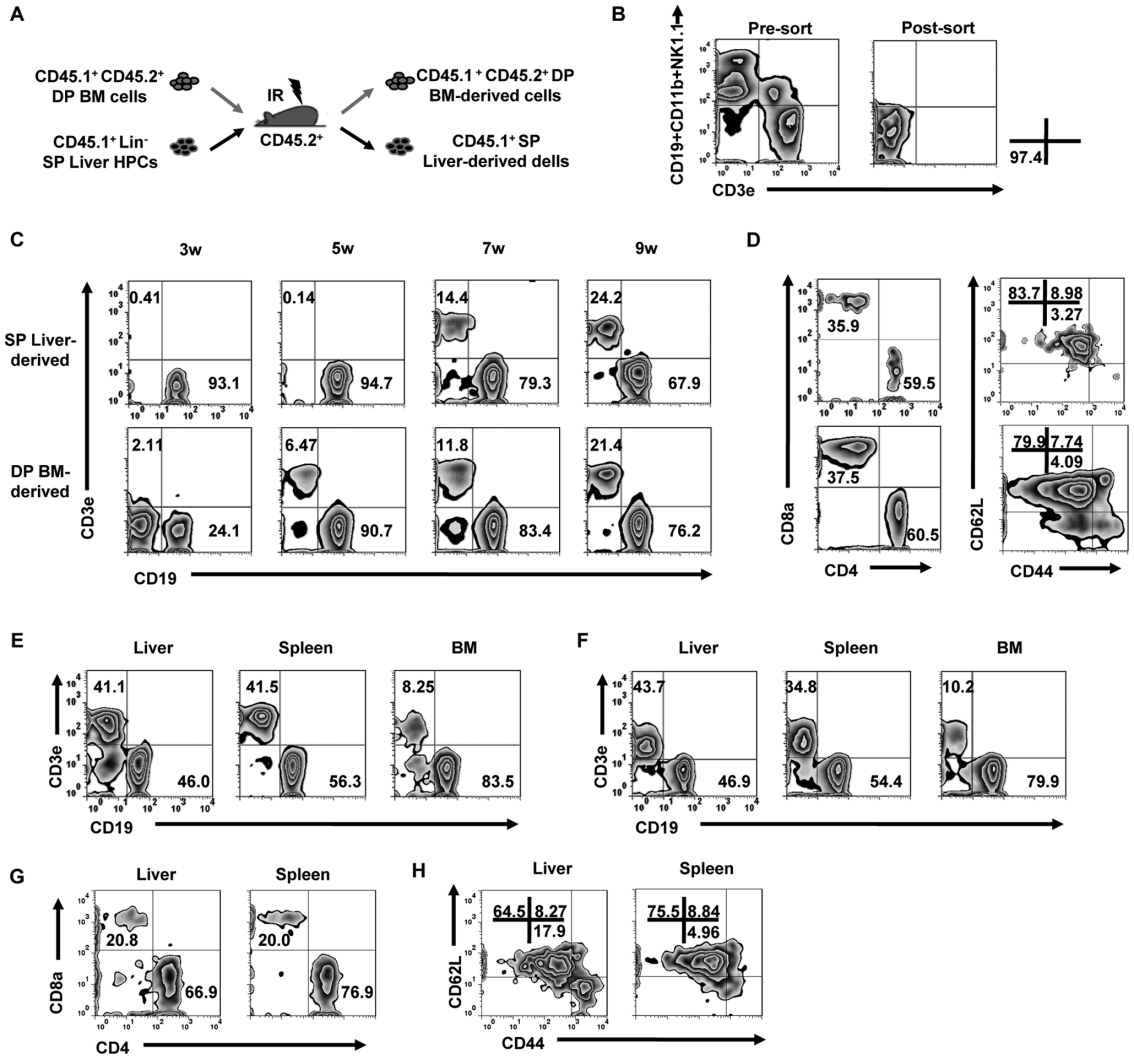
Comparison of the lymphoid reconstitution capacity between liver HPCs and BM in chimeric mice. (A) CD45.1^+^ (SP) liver HPCs (10^5^) were mixed with 10^6^ CD45.1^+^CD45.2^+^ (DP) BM cells and transferred into lethally irradiated CD45.2^+^ B6 mice. (B) The purity of the sorted liver HPCs were evaluated from the pre- and post-sort, as indicated. (C) At the indicated time points after transfer, SP or DP cells were gated from PBMCs to analyze CD3^+^ T cells/CD19^+^ B cells (n = 3 mice/group). (D) CD3^+^ T cells that were gated from cells in (C) at the 7 wk time point were analyzed for CD4/CD8 and CD44/CD62L expression. (E) MNCs also were separated from liver, spleen, and BM to analyze CD3 and CD19 on CD45.1^+^ cells (n = 3 mice/group). (F) Liver HPCs from CD45.1^+^ mice (10^5^) were transferred into *Rag-1^−/−^Il2rg^−/−^* mice. The expression of CD19 and CD3e on CD45.1^+^ cells were analyzed in multiple organs 2 months post-transfer (n = 3 mice/group). (G–H) Analysis of CD4/CD8 and CD44/CD62L expression on CD3^+^ T cells gated from (E) (n = 3 mice/group). All data are representative of 2 independent experiments.

Liver MNCs contain HPCs as well as mature cells [16], [19]. We analyzed the phenotype of donor liver MNCs and BM cells, and found more mature T cells in liver MNCs than in BM cells (Figure S1). In order to evaluate the ability of liver MNC-derived HPCs to generate T cells independently, we first purified liver HPCs by sorting Lin^−^ (CD3^−^CD19^−^NK1.1^−^CD11b^−^) cells from liver MNCs obtained from CD45.1^+^CD45.2^−^ single positive (SP) mice. These purified SP liver HPCs (>95% purity) were co-transferred together with CD45.1^+^CD45.2^+^ double positive (DP) BM support cells into lethally irradiated recipients (Fig. 6A–B). Using this experimental setup, we were able to compare lymphopoiesis from liver HPCs and BM in a same individual by tracking SP- and DP-derived cells, respectively.

We tracked the reconstitution of T and B cells from the 2 different origins in the PBMCs of these chimeric mice. Unlike the results obtained from reconstitution using the whole liver MNC population, we found that T cells were not the major lymphoid subpopulation reconstituted by SP liver HPC-derived lymphopoiesis and that the recovery of SP liver HPC-derived B cells was similar to DP BM-derived B cells. However, the kinetics of reconstitution differed between liver HPCs and BM cells, as SP T cells appeared 2 weeks later than DP T cells (Fig. 6C). Two months after transfer, SP liver HPC-derived T and B cells were recovered in the livers, spleen, and BM (Fig. 6E). These results provide evidence that liver-derived progenitor cells are capable of rebuilding both the T and B lymphocyte population frequencies observed from BM progenitor cells. Similar results were also observed in *Rag-1* ^−/−^ *Il2rg* ^−/−^ recipients (Fig. 6F).

Considering that the kinetics of T cell recovery differed between liver HPCs and BM cells, we determined the precise frequencies of various T cell subsets. In PBMCs, we found no significant difference between the T cell subsets derived from liver HPCs or BM, where a higher frequency of CD4^+^ T cells than CD8^+^ T cells was observed from both transplanted cells; additionally, the majority of these T cells were identified as CD44^low^CD62L^+^ naïve cells (Fig. 6D). The same results were observed in liver and spleen 2 months after transplantation (Fig. 6G–H). Thus, although the kinetics of liver HPC-derived T cell reconstitution was slower than BM, the outcome of reconstitution was similar between the two.

### Liver-resident CD8^+^ T cells in recipients arose from mature T cells in donor liver MNCs

**Figure 7:**
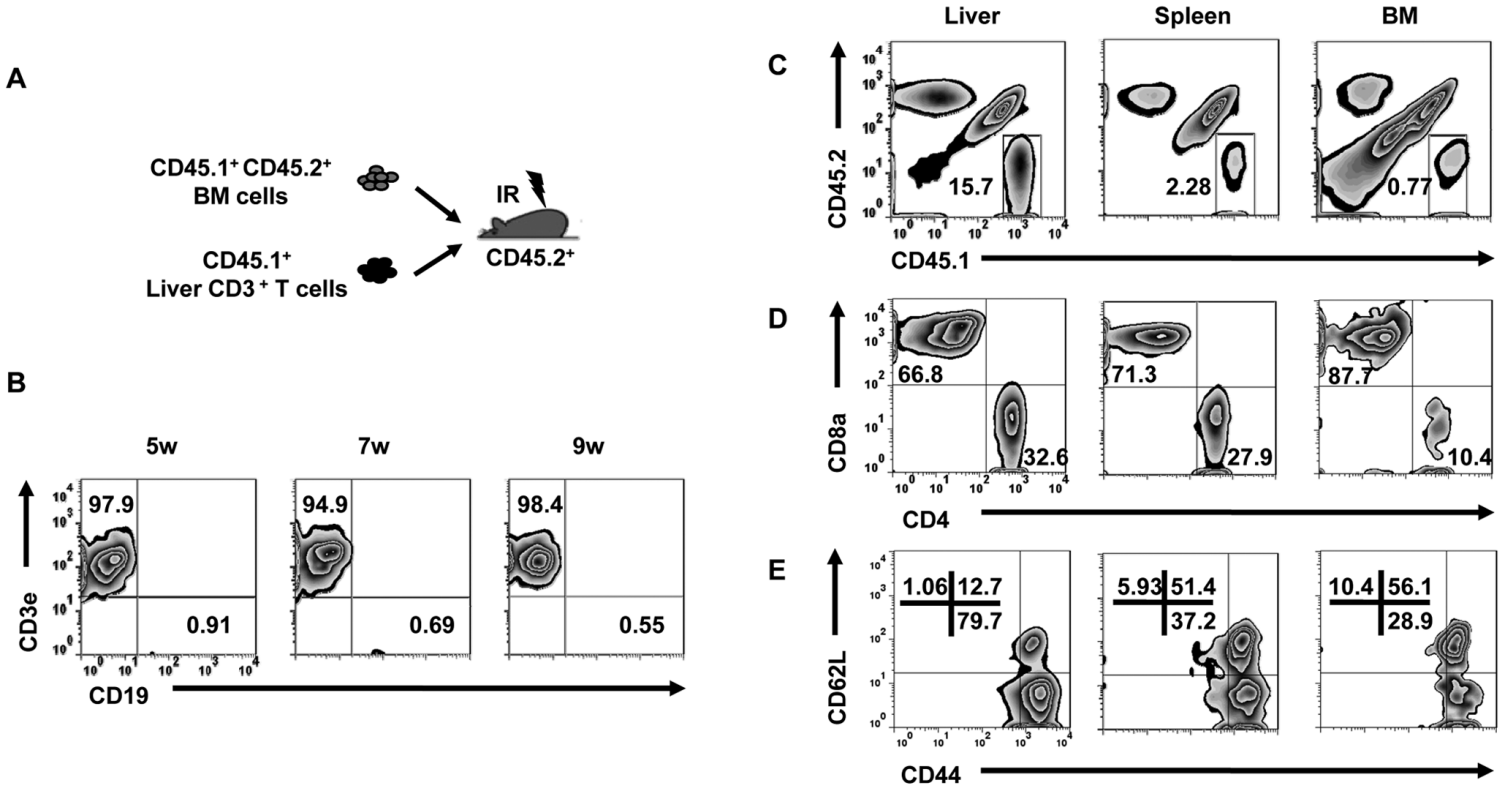
Liver MNCs in recipients contained liver-resident T cells from donor mature T cells. (A) CD3^+^ T cells (10^5^) purified from the livers of CD45.1^+^ (SP) mice were mixed with 10^6^ support BM cells from CD45.1^+^CD45.2^+^ (DP) mice and transferred into lethally irradiated CD45.2^+^ B6 mice. (B) CD45.1^+^ SP cells were gated from PBMCs to analyze the expression of CD19 and CD3e at the indicated time points after transplantation (n = 3 mice/group). (C) MNCs from multiple organs were gated to analyze CD45.1 and CD45.2 expression 2 months after transfer (n = 3 mice/group). (D–E) CD4/CD8 or CD44/CD62L expression on CD45.1^+^ SP cells was analyzed from liver, spleen, and BM (n = 3 mice/group). All data are representative of 2 independent experiments.

Comparing the immune systems reconstituted by the total adult liver MNC population with liver HPCs purified from these MNCs, we noted that the immune system regenerated by total liver MNCs mainly consisted of T cells, whereas the liver HPC-derived immune system did not. Moreover, liver MNC-derived cells preferentially resided in the liver (Fig. 5B–C), while liver HPC-derived cells were distributed into various immune organs without organ specificity (Figure S2). Therefore, mature T cells contained in total liver MNCs may be involved in preferentially reconstituting T cells in the recipient liver. To test this, we co-transplanted SP CD3^+^ T cells purified from liver MNCs and DP support BM cells into lethally irradiated recipients (Fig. 7A). The presence of donor-derived SP T cells was detected early after transplantation and was maintained for at least 9 weeks in PBMCs (Fig. 7B). At 2 months post-transplant, we analyzed the organ distribution of SP cells and found that these mature T cells were proportionally higher in the liver compared to the spleen and BM (Fig. 7C); further studies showed that the majority of these SP cells were CD8^+^ T cells and exhibited a CD44^hi^CD62L^−^ or CD44^hi^CD62L^+^ phenotype (Fig. 7D–E). These results not only help to explain the high frequency of T cells we observed after transplanting total liver MNCs but also suggest that liver MNCs contain a subset of liver-resident CD8^+^ T cells.

## Discussion

In this study, we revealed the hematopoietic capacity of adult livers by describing the precise dynamics of myeloid and lymphoid reconstitution after transplanting total liver MNCs or their cellular components into lethally irradiated or lymphocyte-deficient recipient mice. Since lymphopoiesis generates some of the basic cellular components that comprise the immune system, a clear profile of the lymphocytes generated from both mature lymphoid cells and liver HPCs with lymphopoietic potential may help us to not only understand the unique constitution and function of the liver immune system but also provide insight into the donor-derived immune recovery observed in liver transplant patients.

The question of whether liver HPCs possess similar hematopoietic potential as BM HSCs has been debated for many years. Some studies showed that the hematopoietic capacity of the liver was weaker than that of the BM, as the hematopoietic colonies formed by liver progenitors were less than that formed by the same number of BM progenitors [14]. Consistent with this finding, we found that some subsets of blood cells, such as RBCs, PLTs, and NK cells, regenerated at a much slower rate after liver MNC transplantation than after BMT, suggesting the possibility that adult liver contains fewer HPCs than BM. However, Kotton and colleagues believed that the hematopoietic potential of BM and liver HPC would be similar if the liver progenitors could be isolated with sufficiently high purity [16]. In our study, transplanting 3–4×10^6^ liver MNCs rescued survival in irradiated recipients with a survival rate similar to 10^6^ BM cells, indicating that adult liver likely contains 3-4-fold less HPCs than BM.

The liver is an organ that performs many immune functions and contains unique immune cell populations [21], [22]. Previous studies showed that Kupffer cells, which are liver-resident macrophages, were BM-HSC-independent myeloid cells derived instead from the yolk sac [23], providing evidence that liver-specific hematopoietic systems could alter the local immune system. The generation of liver-resident memory NK cells through a unique, BM-independent developmental pathway also supports this viewpoint [24]. Additionally, progenitors recruited from the bloodstream could give rise to hepatic lymphocytes [16], [25]. Taken together, these results suggest that hematopoiesis in the liver from cells derived from multiple sources gives rise to the unique local cell subsets, which may help to explain the liver's unique immune function as one of the first lines of host defense.

Compared to BMT, distinct mechanisms were observed during the recovery of liver-derived T and B cells in chimeric mice after lethal irradiation or in lymphocyte-deficient *Rag-1* ^−/−^ *Il2rg* ^−/−^. Similar to BMT-derived hematopoiesis, T cell generation from liver precursors was delayed as compared to B cell generation. Unlike BMT-mediated hematopoiesis, however, a robust expansion in mature T cells was observed in recipient livers after liver MNC transplantation. This suggested that proliferating T cells from the transplanted liver MNCs were the dominant liver MNC-derived population in recipient livers, especially in the early days after transfer. In contrast, mature B and NK cells became exhausted shortly after transplantation, but B cells were almost completely recovered by the progenitors within 3 weeks.

Donor liver-derived T cell bias was reported in orthotopic mouse liver transplantation as well, but more CD4^+^ T cells rather than CD8^+^ T cells were observed [26]. It may due to the irradiation in donors eliminating mature CD8^+^ T cells in their system. With regards to liver organ transplantation, we also noted with interest that T cells that proliferated from hepatic CD8 T cells were preferentially present within the liver. Considering that immune cells are the most important effectors in organ rejection, this subset of donor-derived cells may play a role in liver transplantation tolerance.

## Supporting Information

Figure S1 **Phenotypic analysis of donor liver MNCs and BM cells.** Representative dot plots showed percentages of T cells and B cells in the liver and BM. CD4/CD8 or CD44/CD62L expression was further analyzed on T cells (n = 3 mice/group).
(TIF)

Figure S2 **Comparison of liver MNC- or BM-derived cells in chimeric mice.** (A) PBMCs from mice in Fig. 6A were gated to analyze the CD45.1 and CD45.2 expression at the indicated time points after transfer (n = 3 mice/group). (B) MNCs from multiple organs were gated to analyze CD45.1 and CD45.2 expression 2 months after transplantation (n = 3 mice/group). Data are representative of 2 independent experiments.
(TIF)
