# Supplementary figures and images for: Characterizing the Lymphopoietic Kinetics and Features of Hematopoietic Progenitors Contained in the Adult Murine Liver In Vivo
Source: PLoS One. 2013 Oct 9;8(10):e76762. doi: 10.1371/journal.pone.0076762 (PMC3793923; doi:10.1371/journal.pone.0076762)

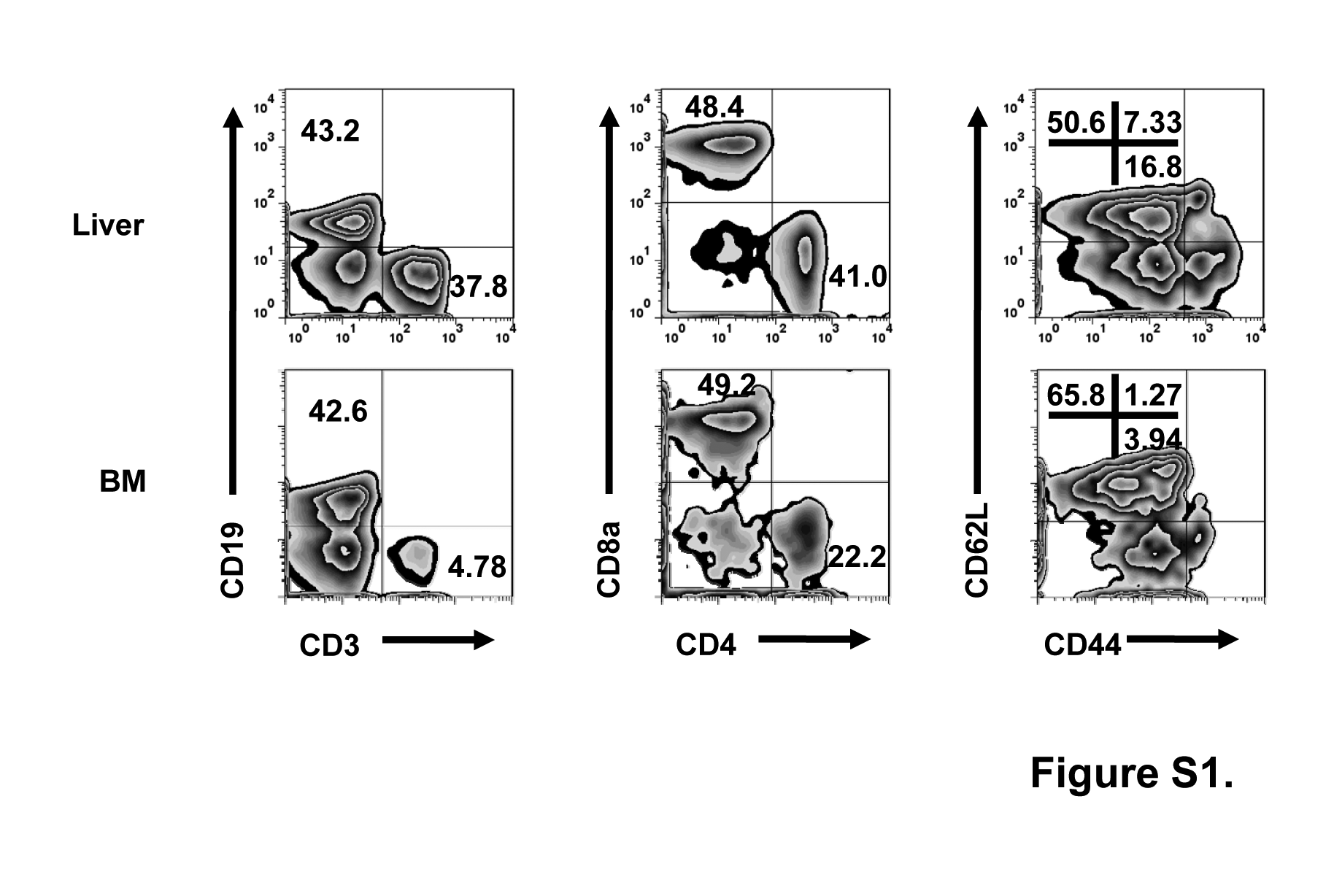

Supplement: Figure S1 — Phenotypic analysis of donor liver MNCs and BM cells. Representative dot plots showed percentages of T cells and B cells in the liver and BM. CD4/CD8 or CD44/CD62L expression was further analyzed on T cells (n = 3 mice/group). (TIF) [file pone.0076762.s001.tif]

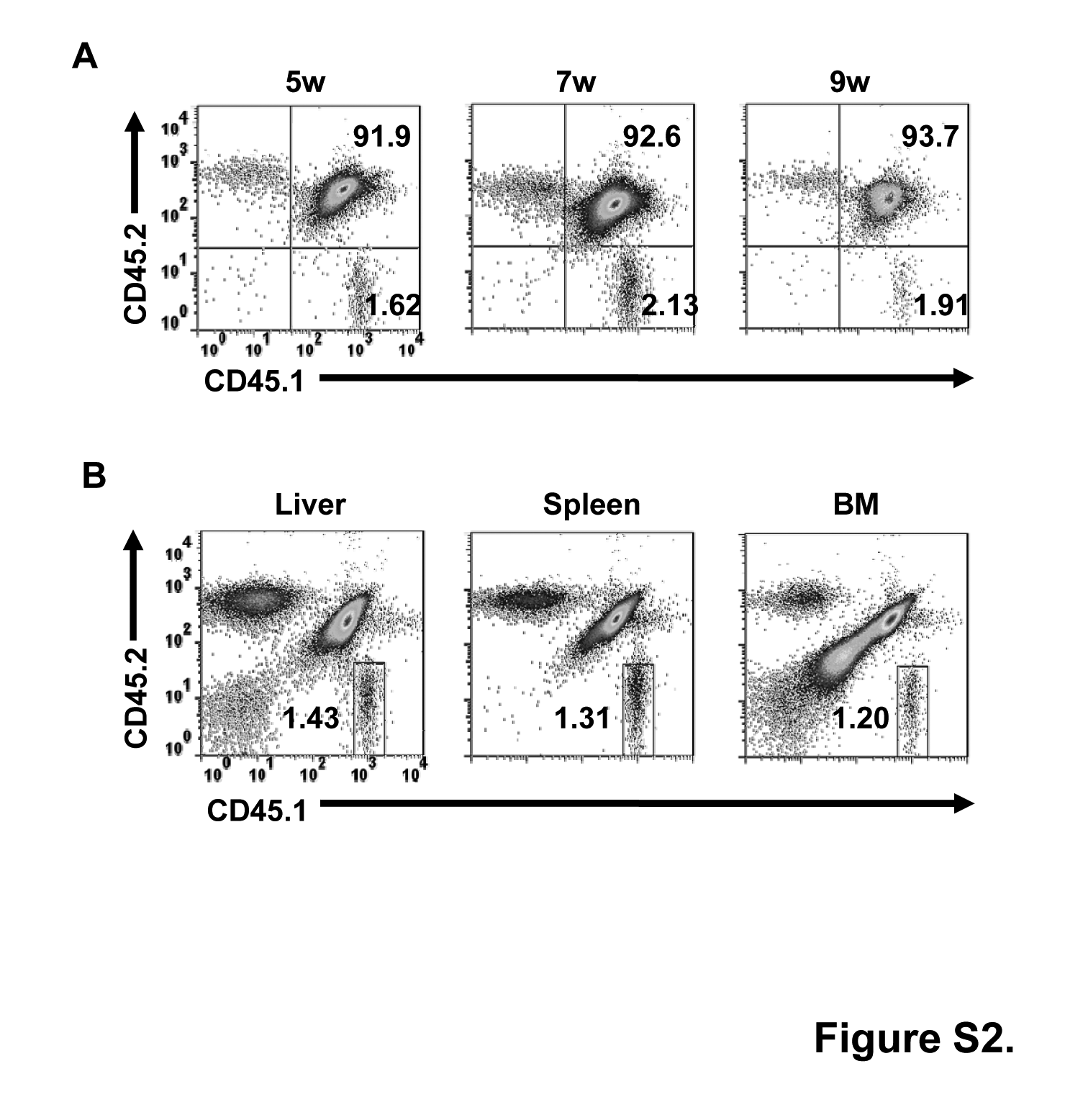

Supplement: Figure S2 — Comparison of liver MNC- or BM-derived cells in chimeric mice. (A) PBMCs from mice in Fig. 6A were gated to analyze the CD45.1 and CD45.2 expression at the indicated time points after transfer (n = 3 mice/group). (B) MNCs from multiple organs were gated to analyze CD45.1 and CD45.2 expression 2 months after transplantation (n = 3 mice/group). Data are representative of 2 independent experiments. (TIF) [file pone.0076762.s002.tif]
